# Analysis of ultrastructure and microstructure of blackbird (*Turdus merula*) and song thrush (*Turdus philomelos*) eggshell by scanning electron microscopy and X-ray computed microtomography

**DOI:** 10.1038/s41598-022-16033-5

**Published:** 2022-07-13

**Authors:** Krzysztof Damaziak, Agata Marzec

**Affiliations:** 1 Department of Animal Breeding, Institute of Animal Science, Warsaw University of Life Sciences—SGGW, Ciszewskiego 8, 02-786 Warsaw, Poland; 2 Department of Food Engineering and Process Management, Institute of Food Science, Warsaw University of Life Sciences—SGGW, Nowoursynowska 159c, 02-776 Warsaw, Poland

**Keywords:** Physiology, Structural biology, Zoology

## Abstract

The unique structure of the egg allows for efficient reproduction on land. Although the functions of the egg are ensured by the concomitant cooperation of all its structures, the eggshell also plays a significant role. Apart from maintaining an aqueous environment within the egg along with controlled gas exchange, the color and pigmentation pattern of eggshell contributes to identification and protection. As a result of all these functions, the structure, shape, and pigmentation of eggshell greatly vary across the class of birds, and understanding these three variability-determining factors may aid in better interpretation of evolutionary mechanisms. In this study, we analyzed for the first time the structure, mineral composition, and characteristics of the pigmentation of blackbird (*Turdus merula*) and song thrush (*Turdus philomelos*) eggshells. The shell of blackbird eggs is much thicker compared to the shell of song thrush eggs which is due to a much thicker crystalline and palisade layers. In both species, strongly elongated mammillary knobs are observed, which create a large space between the mineralized shell and the egg membranes. The blackbird egg shell has a higher water vapor conductivity which is due to the larger diameter of the circle and the surface area of individual pores. The primary compound entering the mineral composition of the shell in both species is CaCO_3_ however, the thrush egg shells contained more Mg in all layers except the crystalline layer, and S in the crystalline and palisade layers. The two species clearly differ in the size and distribution of pigment spots on the eggshell. We suppose that the differences in shell structure and pigmentation presented in this study may in the future provide a basis for explaining the reasons for the much lower reproductive efficiency of song thrush compared to blackbird.

## Introduction

Colonization of terrestrial habitats by vertebrates began around 360 million years ago. This was possible due to the development of amniotic egg which is considered to be an important evolutionary process. Most sauropsids (reptiles and birds), as well as some of the primitive mammals (monotremes), were able to create an aqueous environment for embryogenesis on land owing to the layered structure of their membranes and biomineral eggshell^1^. Birds are the only class of vertebrates, in which all species are oviparous, and their eggs possess a hard shell with a specific structure, adapted to key reproductive functions^2^. The most important of these functions are the preservation of aqueous environment within the egg while maintaining regulated gas exchange, protection against microbial and physical factors from the external environment, and supply of calcium for the developing embryo after its yolk resources are depleted^2^. These functions are ensured by the structure of the eggshell and chemical compounds present in it.

The general scheme of the structure and chemical composition of eggshell is comparable for all avian species, at least for those that have been investigated so far^3–5^. The outermost layer of eggshell is made of two white-side membranes consisting of tightly interwoven fibers. Of these two membranes, the internal one remains uncalcified, whereas the external one undergoes partial mineralization, serving as a matrix for the initiated calcification process and acting as an anchoring point for the pseudoperiodically distributed and organic substance-rich mammillary knobs (Mk)^6^. These poorly understood eggshell fragments play an important role in calcium mobilization and the formation of the mammillary layer (Ma). At this stage, the eggshell pores are already starting to develop^7^. Ma is a relatively thin layer forming spherolithic aggregates of calcite crystals appearing as reversed cones, which unite to form a compact palisade layer (Pa)^8^. Pa is the thickest element of eggshell and combines with the outermost crystalline layer (Cr), which is protected by a cuticle (Ct). Ct contains hydroxyapatite crystals^8^ and most of the pigment^9^. The entire eggshell is porous, and pore channels are found between the Ma cones, extending radially across Pa reaching to the exterior^10^. Thus, these channels penetrate all levels of the eggshell, enabling the exchange of water and metabolic gases^6^.

In chemical terms, avian eggshell is mainly made of trigonal calcium carbonate phase (CaCO_3_), which is referred to as calcite (approximately 95%), and organic matrix (about 3.5%)^2,11^. The other components found are minerals, including phosphorus (P), potassium (K), sodium (Na), and magnesium (Mg), as well as iron (Fe), zinc (Zn), copper (Cu), and aluminum (Al) in trace amounts^12^.

Although numerous studies have confirmed that the structural scheme of eggshell is comparable among avian species, the eggshell structure of a majority of species remains to be investigated. Thus far, the eggshell structure of domesticated species has been widely studied^5,7,13^. However, the variability of this trait among wild birds has received less attention^3,14,15,17–19^. Studies have proven that the evolutionary adaptation of birds to specific environmental conditions is determined by the variability of the eggshell structure. The best example here is the unique porosity pattern of Malleefowl eggshell, which allows for better gas conductivity and efficient gas exchange compared to other species, and is critical for normal development in the underground environment^3^. The study by Board and Tullett^14^ and by Szczerbińska and Wiercińska^15^ showed that an additional reticular layer is found over Pa in emu and cassowary eggshell. This layer, which is specific for cassowaries, is highly porous and serves in air storage during incubation^16^. Another interesting finding is the specificity of the surface structure of the guillemot eggshell. Guillemot are marine birds that nest on unprotected cliffs. Their eggshell has unique cone-shaped nanostructures which cause saltwater drops to quickly form the shape of a ball and separate from the egg. As a result, the eggs of guillemot are never humid, which facilitates their spontaneous cleaning of feces and residues of crushed rocks^17,18^.

Indeed, the above-cited examples represent only a small portion of what is known about the interspecific variability of avian eggshell. A number of individual traits of eggshell remain unexplored due to a wide range of species as well as the dynamic evolutionary process. Therefore, the ultrastructural and microstructural characteristics of the eggshells of two wild bird species of the family Turdidae: blackbird (*Turdus merula*) and song thrush (*Turdus philomelos*) were analyzed. To the best of our knowledge, this is the first study on eggshells of these birds. The study was conducted using scanning microscopy and x-ray microtomography, which is still not a widely used method. Due to cost and analysis time, most studies rely on microscopic techniques to examine shell structure. Computed microtomography is a technique that produces very large amounts of high-resolution images. These images are used to generate digital three-dimensional models of samples. This technique made it possible to determine the porosity of the shells. In addition, the novelty and importance of this study is also due to the elucidation of the mineral composition of shells, which affects the taxonomy and evolution of species.

## Results

### Ultrastructure of blackbird and song thrush eggshell

**Figure 1:**
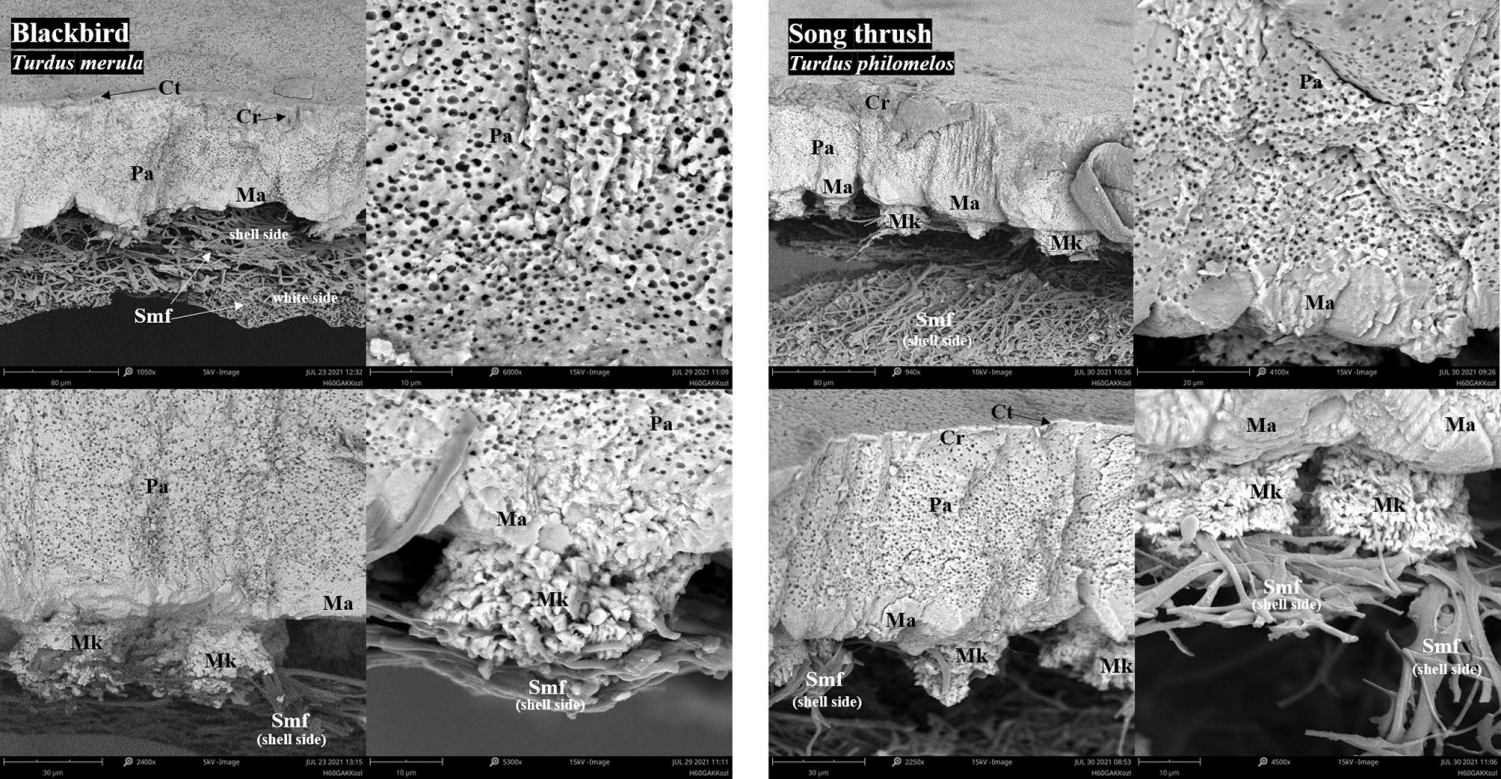
SEM micrograph showing the cross section of the eggshell of blackbird (*Turdus merula*) and song thrush (*Turdus philomelos*). Cuticle (Ct), crystalline layer (Cr), palisade layer (Pa), mammillary layer (Ma), mammillary knobs (Mk), and shell-side or white-side membrane fibers (smf) are evident.

SEM micrographs revealing the cross-section morphology of the eggshell of blackbird and song thrush are presented in Fig. 1. The eggshell of both species showed a similar structural pattern and consisted of four main layers: Cr (with thin Ct coating), Pa, Ma, and Mk.

**Figure 2:**
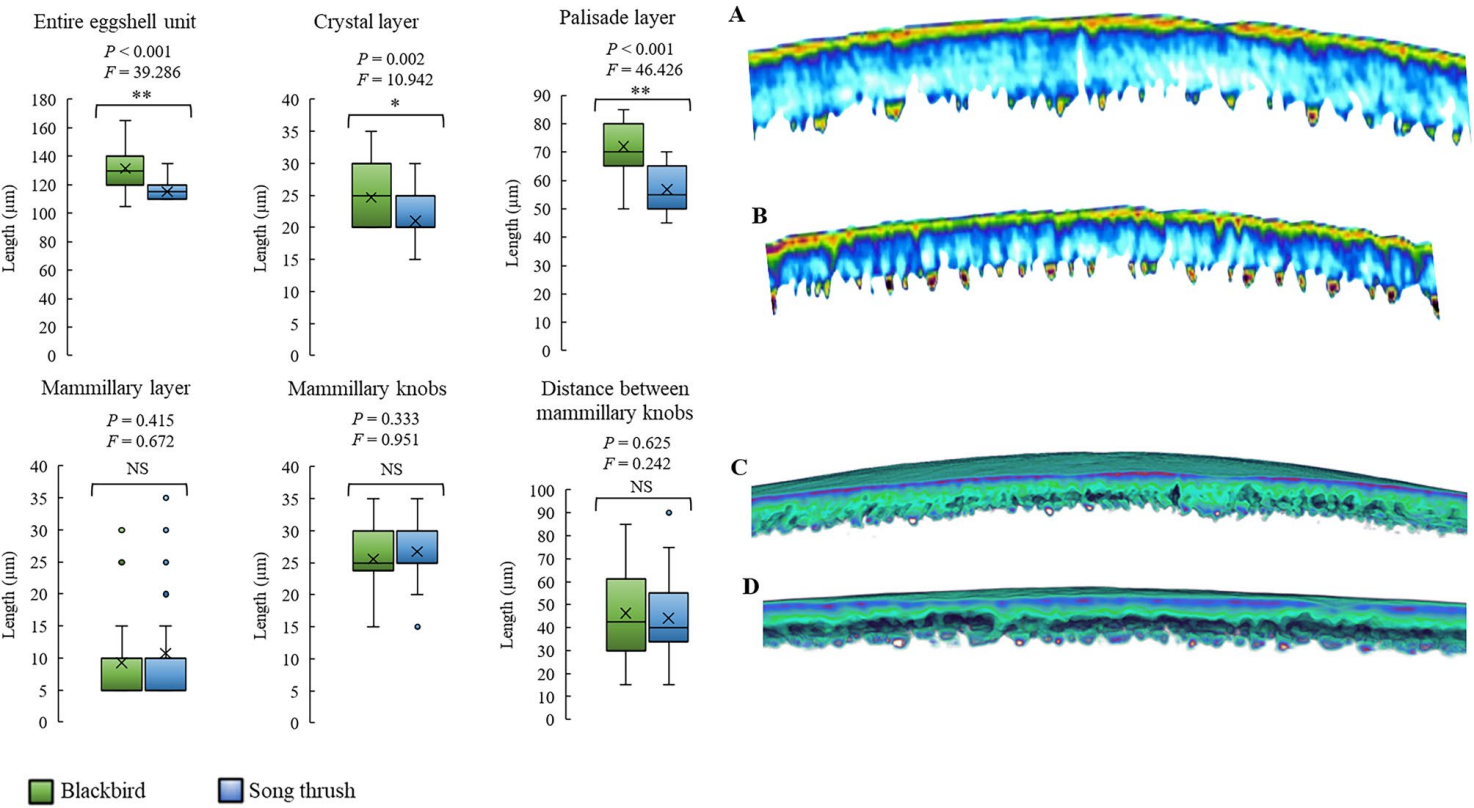
Comparison of the dimensions of individual layers present in the eggshell of blackbird (*Turdus merula*) and song thrush (*Turdus philomelos*). Box plots show the means (cross), standard deviation (box height), and the low and high range (vertical bars). 2D (**A** and **B**) and 3D (**C** and **D**) scans of the eggshell of blackbird and song thrush. Significant differences determined by ANOVA: **P < 0.01; *P < 0.05; NS (no significant differences, *P* > 0.05).

Compared to the eggshell of song thrush, the whole eggshell of blackbird was thicker (*P* < 0.001), but this may be related to the thicker Cr (*P* = 0.002) and Pa (*P* < 0.001). Ma and Mk did not differ in their thickness (*P* = 0.415 and *P* = 0.333, respectively) (Fig. 2). Similarly, no differences were found in the distance between singular Mk (*P* = 0.625) (Fig. 2).

**Figure 3:**
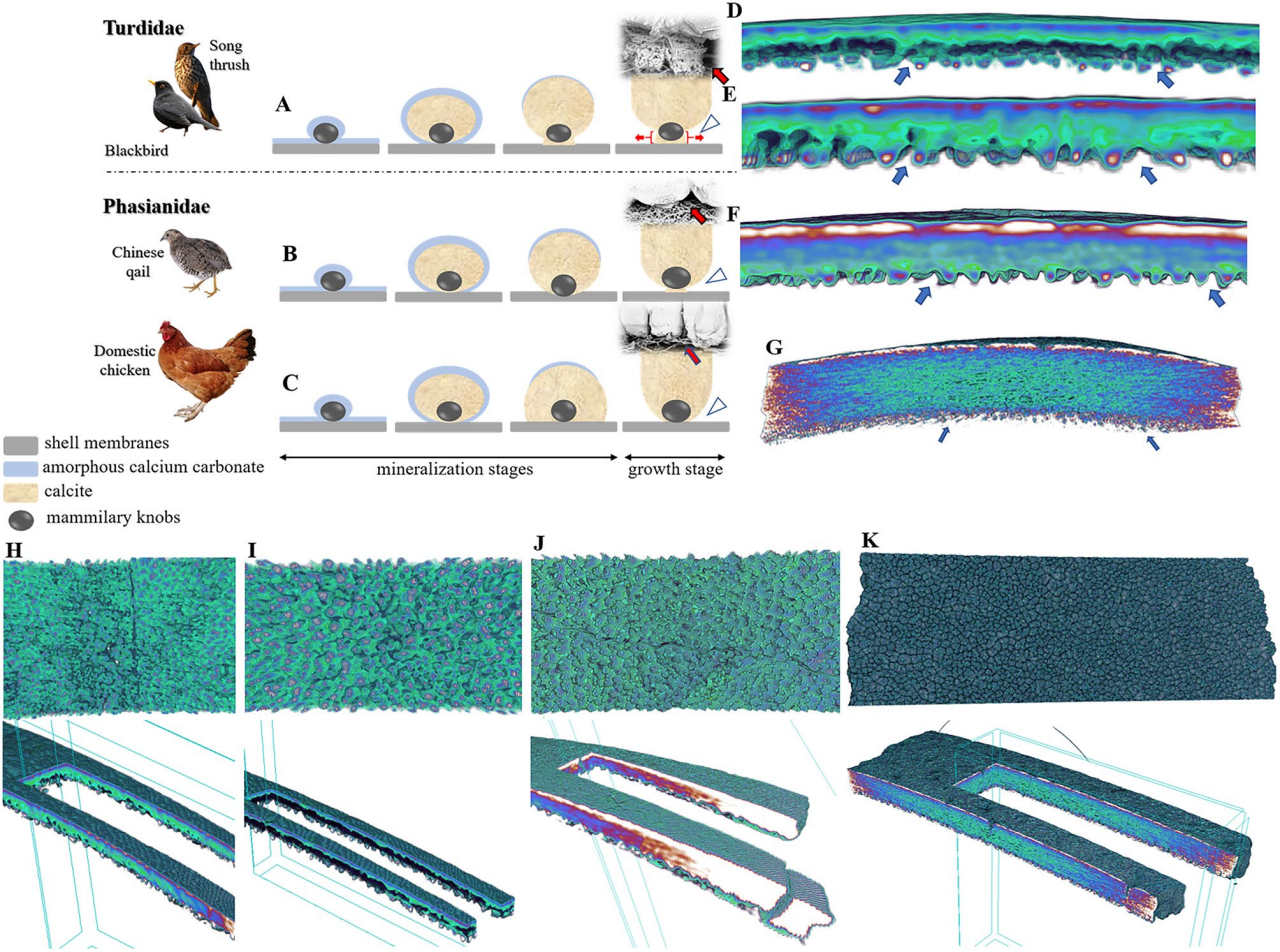
Comparison of the eggshell structure of blackbird (*Turdus merula*), song thrush (*Turdus philomelos*), Chinese (king) quail (*Synoicus chinensis*), and domestic chicken (*Gallus gallus*). Schematic representation illustrating the process of mineralization around mammillary knobs of Turdidae: blackbird and song thrush (**A**), and representatives phasianidae bird family: Chinese quail (**B**) and domestic chicken (**C**). The schema was made based on Le Roy et al.^11^ The 3D scans reveal the cross section (**D**–**G**) and the inner surface of the eggshell (**H**–**K**). The lower scans (**H**–**K**) show the cross-section structure at two points of the eggshell simultaneously. (**D**, **H**): blackbird; (**E**, **I**): song thrush; (**F**, **J**): Chinese quail; (**G**, **K**): domestic chicken. All arrows indicate the spaces formed between Mk.

A comparative analysis of the eggshell structure of blackbird, song thrush, domestic chicken, and Chinese quail (Fig. 3) indicated the similarity of the main eggshell units and Mk morphology between blackbird, song thrush, and Chinese quail. In contrast, singular eggshell units of domestic chicken differed in shape, and Mk were mainly hidden within Ma (Fig. 3).

**Figure 4:**
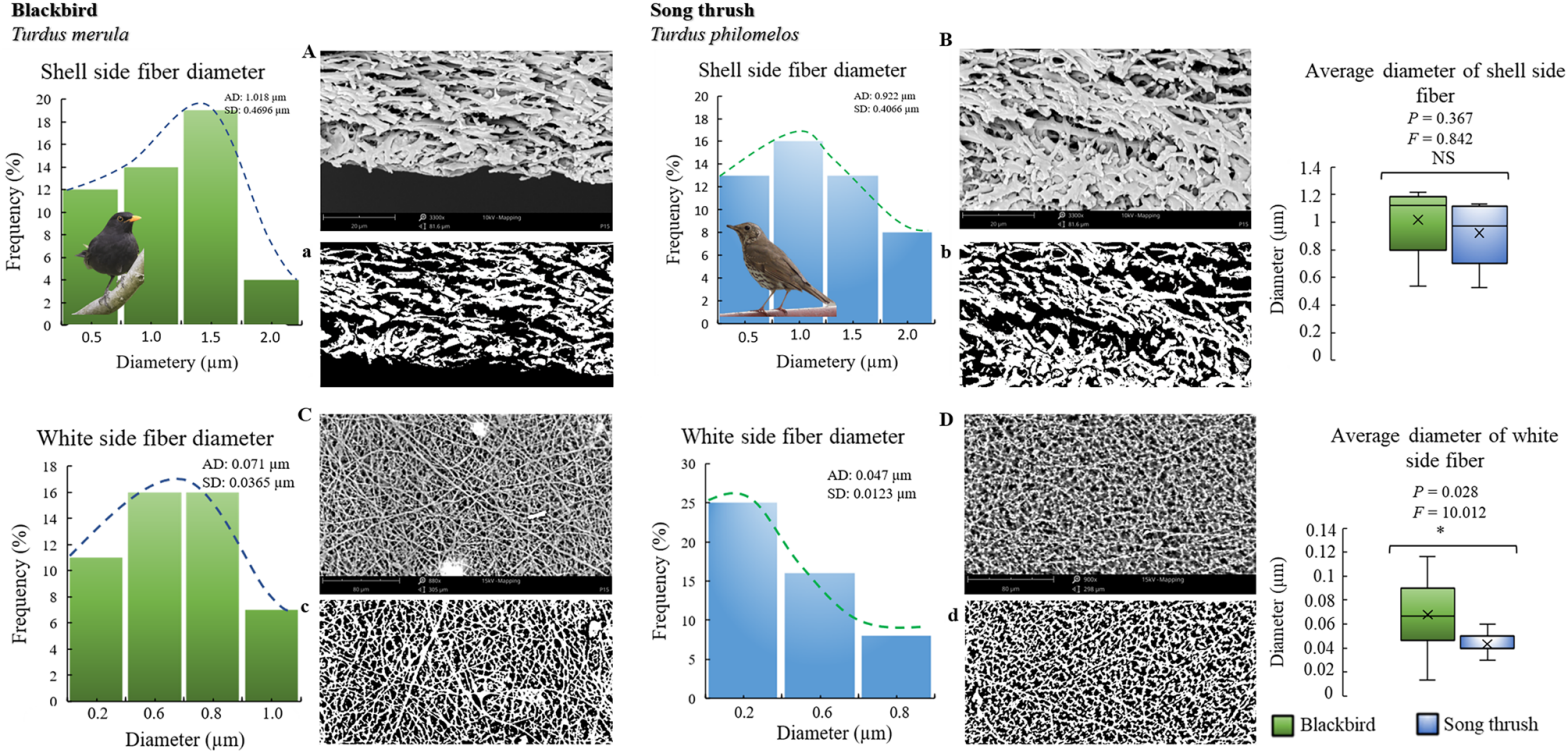
Variability and diameter of the shell-side and white-side membrane fibers of blackbird and song thrush eggshell, based on SEM micrographs (**A**–**D**) and binary image (a-d) (MultiScanBase v. 18.03). The histograms show the diameter distribution of the fibers (AD: average diameter, SD: standard deviation). Box plots show the means (cross), standard deviation (box height), and the low and high range (vertical bars) of fiber diameter. Significant differences by ANOVA: *P < 0.05; NS (no significant differences, *P* > 0.05).

The diameter of outer shell membrane fibers present in the eggs of both blackbird and song thrush was found to be similar (*P* = 0.367), with a similar distribution from 0.5 to 2.0 µm. The inner shell membrane fibers were thicker in blackbird compared to that in song thrush (*P* = 0.028) (Fig. 4).

### Ultrastructure, microstructure, and water vapor conductance of blackbird and song thrush eggshell

**Figure 5:**
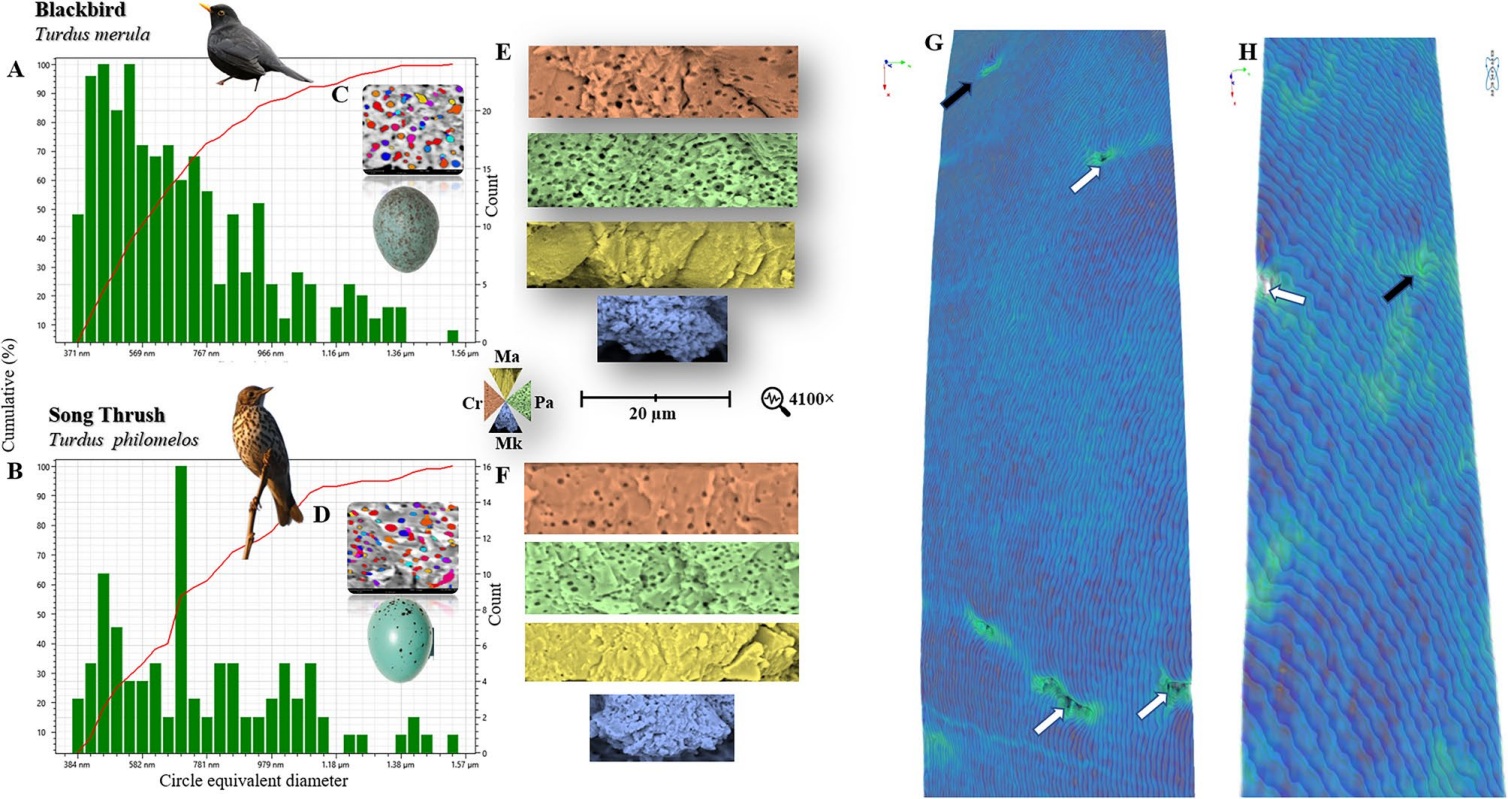
Porosity of blackbird and song thrush eggshell. Histograms (**A**) and (**B**) show the distribution of pores of different diameters as measured by PoroMetric with Phenom scanning electron microscope (images **C** and **D**). The SEM micrographs (**E**) and (**F**) (animated and colored) show the differences in the porosity of crystalline layer (Cr), palisade layer (Pa), and mammillary layer (Ma) as well as the ultrastructure of mammillary knobs. The 3D scans (**G**) and (**H**) show the surface of the eggshells of blackbird and song thrush, indicating open (white arrows) and closed porosity (black arrows).

The analyses of ultrastructure parameters using SEM (Table 1, Fig. 5) and 3D microstructure using X-ray computed micrography (Table 2, Fig. 5) did not reveal any significant differences between the eggshells of blackbird and song thrush.

**Table 1:** Ultrastructure parameters of blackbird and song thrush eggshell determined using PoroMetric with Phenom scanning electron microscope.

Parameters	Blackbird	Song thrush	ANOVA		
			SEM	*F*	*P*-value
Average object area-equivalent circle diameter (nm)	7.36 × 10^–7^ ± 2.63 × 10^–7^ [3.71 × 10^–7^–1.56 × 10^–6^]	7.46 × 10^–7^ ± 2.57 × 10^–7^ [3.70 × 10^–7^–1.57 × 10^–6^]	2.05 × 10^–7^	0.128	0.720
Circumference (µm)	2.65 × 10^–6^ ± 9.85 × 10^–7^ [1.21 × 10^–6^–5.78 × 10^–6^]	2.74·10^–6^ ± 1.01 × 10^–6^ [1.20 × 10^–6^–6.04 × 10^–6^]	7.84 × 10^–8^	0.684	0.409
Area (nm)	4.80 × 10^–7^ ± 3.58 × 10^–7^ [1.08 × 10^–7^–1.91 × 10^–6^]	4.89 × 10^–7^ ± 3.52 × 10^–7^ [1.07 × 10^–7^–1.94 × 10^–6^]	2.78 × 10^–8^	0.059	0.809
Volume by area (nm^3^)	2.97 × 10^–13^ ± 3.41 × 10^–12^ [2.67 × 10^–14^–1.99 × 10^–12^]	3.02 × 10^–13^ ± 3.43 × 10^–12^ [2.65 × 10^–14^–2.04 × 10^–12^]	2.69 × 10^–14^	0.020	0.889

Values are means and errors for 10 eggshells of blackbird and 10 eggshells of song thrush (each for different egg).

Statistically significant at *P* < 0.05.

**Table 2:** 3D microstructure parameters of blackbird and song thrush eggshell.

Parameters	Blackbird	Song thrush	ANOVA		
			SEM	*F*	*P*-value
Object volume (mm^3^)	1.45 × 10^–3^ ± 0.001 [4.54 × 10^–4^–2.96 × 10^–3^]	1.35 × 10^–3^ ± 0.001 [1.95 × 10^–4^–2.85 × 10^–3^]	0.0003	0.033	0.858
Percent object volume (%)	42.31 ± 25.66 [13.28–86.67]	39.20 ± 30.34 [5.70–83.47]	26.69	0.040	0.844
Object surface (mm^2^)	0.215 ± 0.06 [0.122–0.293]	0.222 ± 0.10 [0.087–0.322]	0.22	0.028	0.870
Object surface/volume ratio (mm^–1^)	182.22 ± 137.69 [98.95–281.48]	247.95 ± 70.80 [108.96–446.45]	107.56	1.230	0.291
Object surface density (mm^–1^)	62.87 ± 16.47 [35.66–85.76]	64.47 ± 30.47 [25.43–94.41]	22.87	0.015	0.906
Euler number (–)	10.14 ± 8.14 [–14–55]	5.67 ± 9.81 [–33–33]	6.06	0.126	0.730
Fractal dimension (–)	1.54 ± 0.09 [1.42–1.65]	1.51 ± 0.15 [1.26–1.67]	0.033	0.121	0.734

Among the 2D microstructure parameters determined using X-ray computed microtomography, the average object area-equivalent circle diameter and the mean individual pore area were found to differ significantly between the two studied species (*P* < 0.001) (Table 3). The pores of blackbird eggshell had a higher circle diameter and mean individual pore area (by 14% and 16%, respectively). A higher number of channels (N) were detected on the surface of blackbird eggshell compared to that of song thrush eggshell (*P* = 0.043). The tested eggshells also showed differences in their thickness, which was treated as pore length. Therefore, the eggshell porosity (Ap/Ls) (*P* < 0.001) and water vapor conductance (*P* < 0.001) were found to differ between the studied species.

**Table 3:** 2D microstructure parameters and water vapor conductance of blackbird and song thrush eggshell.

Parameters	Blackbird	Song thrush	ANOVA		
			SEM	*F*	*P*-value
Average object area-equivalent circle diameter	0.042 ± 0.021 [0.006–0.080]	0.036 ± 0.021 [0.006–0.079]	0.0004	24.70	< 0.001
A—mean individual pore area (mm^2^)	0.0017 ± 0.0015 [0.00003–0.005]	0.0011 ± 0.0013 [0.00003–0.005]	2 × 10^–6^	22.54	< 0.001
As—surface area of eggshell (cm^2^)	17.55	15.94	–	−	−
N—total number of pores (per egg)	6498 ± 3560 [3510–21,059]	6130 ± 3281 [3187–19,123]	936	4.07	0.043
Ap—total pore area (mm^2^	8.36 ± 6.50 [0.09–48.47]	6.09 ± 4.51 [0.08–15.55]	31.30	52.78	< 0.001
Ls—shell thickness (µm)	131.47 ± 13.63 [105–165]	115.29 ± 6.39 [110–135]	1.82	39.286	< 0.001
Ap/Ls (mm)	63.580 ± 49.43 [0.67–368.65]	52.78 ± 39.14 [0.69–134.86]	987.76	18.17	< 0.001
G_H2O_—water vapor conductance	133.52 ± 103.80 [1.40–774.17]	110.83 ± 82.19 [1.45–283.20]	766.04	18.17	< 0.001
log G_H2O_	1.95 ± 0.46 [0.15–2.89]	1.83 ± 0.53 [0.16–2.45]	0.24	19.90	< 0.001

### Eggshell color and pigmentation

**Figure 6:**
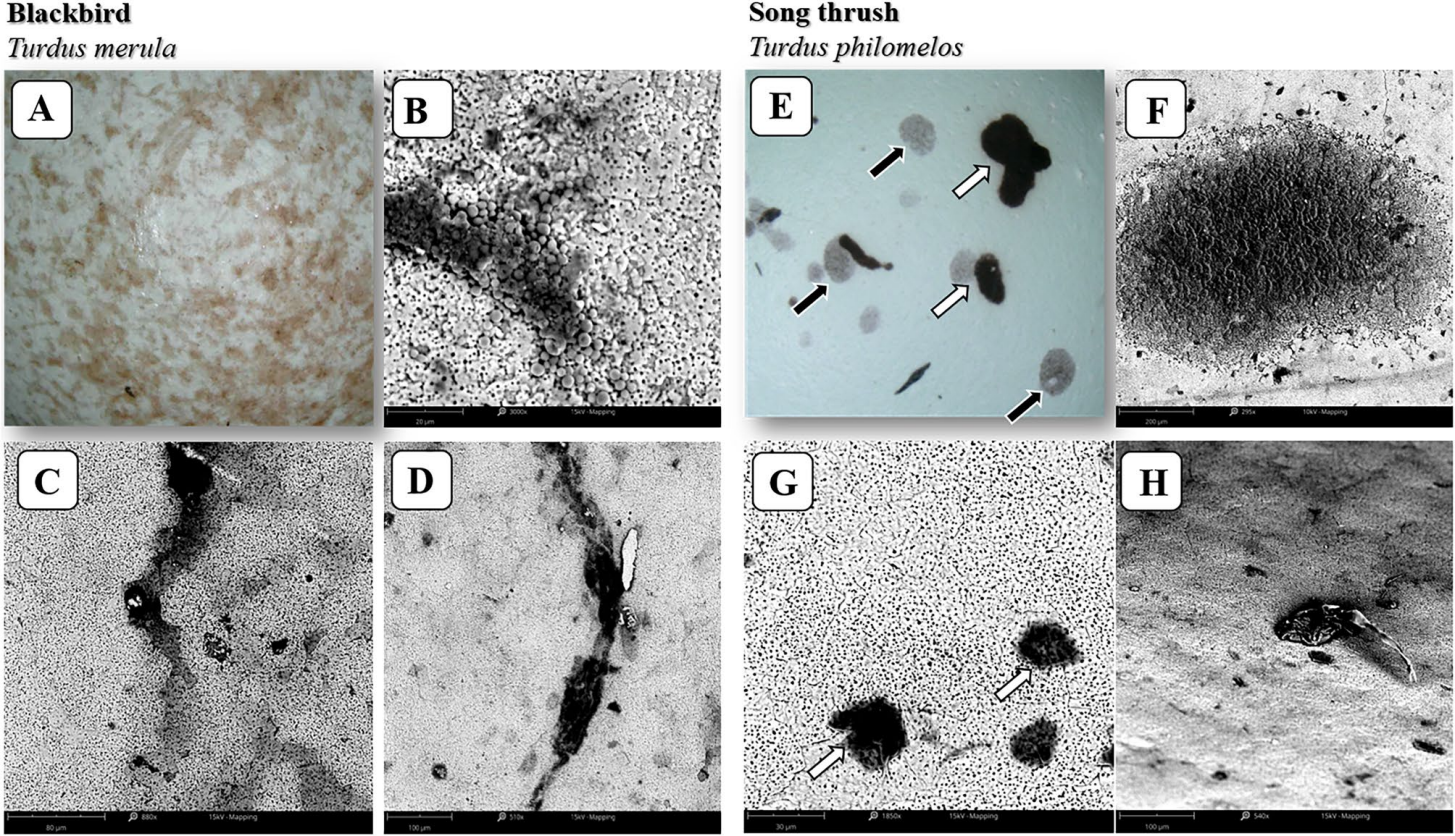
Pigmentation of the eggshells of blackbird and song thrush. Images (**A**) and (**E**) show the actual color of the eggshells. The micrographs show the arrangement and shape of the pigment spots at various magnifications (**B**–**D**: blackbird eggshell, **F**–**H**: song thrush eggshell). Black arrows on the surface of the song thrush eggshell indicate deep pigment spots (**E**), and white arrows indicate surface spots (**F** and **G**).

Figure 6 presents a comparison of the actual color of blackbird (A–D) and song thrush (F–H) eggshell. The SEM micrographs reveal the differences in the pigment spots on eggshells, mainly their shapes and location.

The basic eggshell color of both species was light blue, but in the case of blackbird the eggshell was heavily covered with brown spots, and the surface of the base color thus appeared smaller than that of the pattern. On the contrary, the eggshell of song thrush had a few black pigment spots (mainly on the blunt end), with the base color clearly visible. Pigment spots on the blackbird eggshell were mainly elongated-fusiform (C and D) and found on the outer Ct surface (B). On the other hand, in the case of song thrush, the pigment spots were irregular or oval in shape (F–H) and were of two types. The outer spots were located under the Ct surface on outer Cr (these spots were formed through calcification) and, similar to blackbird, formed after Ct (shortly before egg-laying) (E–G).

### Disparity in eggshell elemental composition

**Figure 7:**
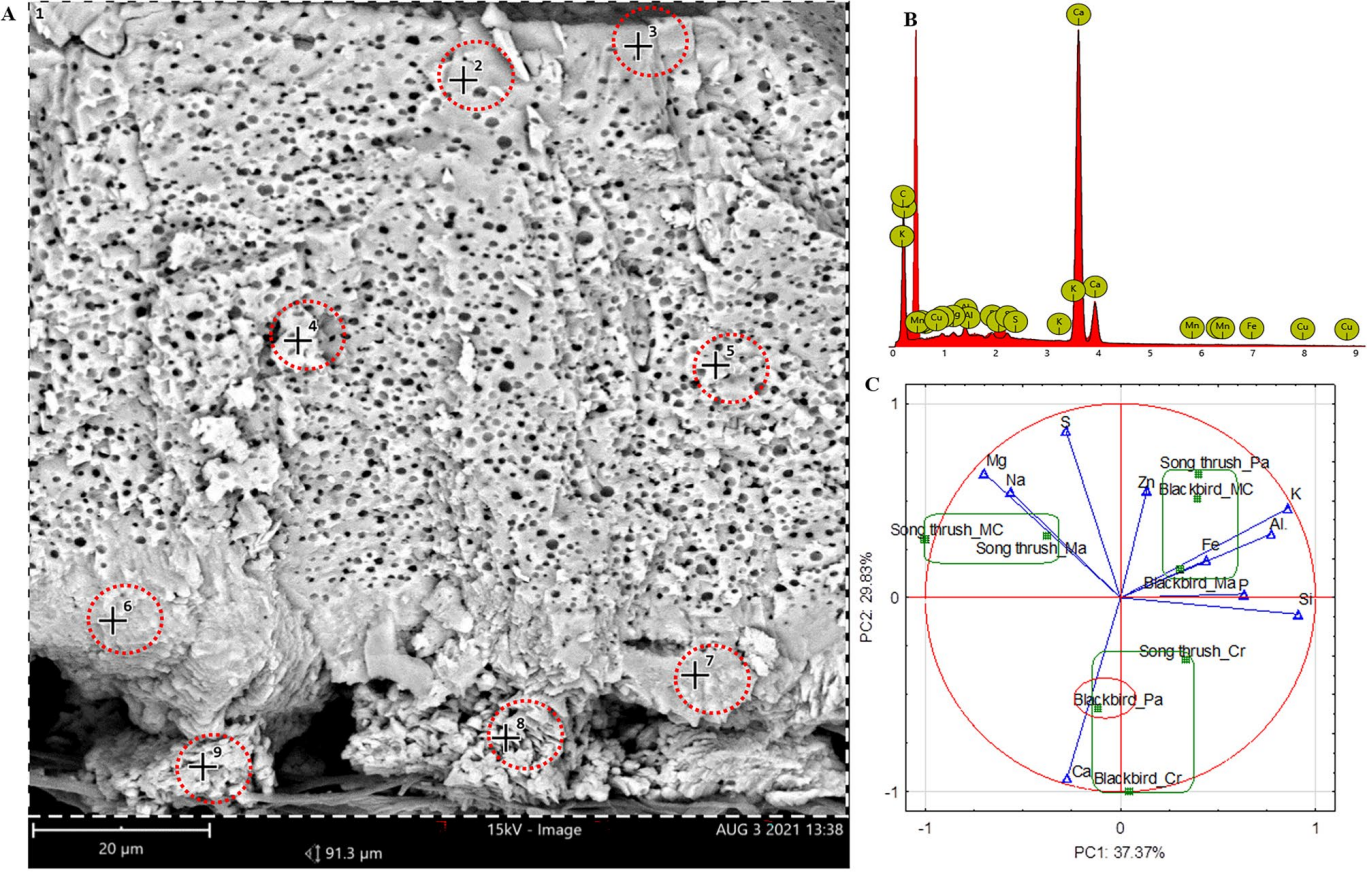
Comparative analysis of elemental content in different layers of blackbird and song thrush eggshells. Micrograph (**A**) shows the measurement sites and (**B**) shows exemplary spectra of the elements from the SEM–EDS analysis. Diagram (**C**) provide the results of PCA, in which PC1 and PC2 are the first two principal components.

The results obtained from the comparative analysis of the elemental composition of specific blackbird and song thrush eggshell are presented in Table 4 and Fig. 7C (PCA). The basic element of all eggshells was Ca (content: 87.55–99.12%), which was found mainly in the form of calcite CaCO_3_. No significant differences in the content of Ca were observed between specific layers within and between the studied bird species (*P* > 0.05). However, the content of Mg (both *P* = 0.000) and S differed depending on the species and eggshell layer (*P* = 0.000 and *P* = 0.024, respectively). Compared to the eggshells of blackbird, song thrush eggshells contained more Mg in all layers, except for Cr, and more S in Cr and Pa. The differences in the content of Si and K (Table 4) may be related to the complete lack or trace amounts (outside of the detection limit) of both elements in Mk of song thrush eggshell.

**Table 4:** Results of the SEM–EDS analysis of elemental content in different layers of blackbird and song thrush eggshell.

Element	Cr—crystalline layer		Pa—palisade layer		Ma—mammillary layer		Mk—mammillary knobs		ANOVA				
	Song thrush	Blackbird	Song thrush	Blackbird	Song thrush	Blackbird	Song thrush	Blackbird	Pooled SEM	*F*/*P*-value	Species	Layer	Species × layer
Ca	96.32 ± 1.22 [94.95–99.12]	97.35 ± 0.80 [96.18–98.64]	95.20 ± 1.23 [93.18–97.71]	96.99 ± 0.63 [95.59–97.95]	95.32 ± 1.05 [93.72–97.19]	95.53 ± 3.13 [88.62–97.98]	96.28 ± 0.73 [95.12–97.66]	95.07 ± 3.01 [87.55–97.84]	0.55	*F*	1.4	2.5	2.7
										*P*-value	0.247	0.068	0.054
Al	0.67 ± 0.24 [016–0.93]	0.54 ± 0.23 [0.20–0.86]	0.82 ± 0.24 [0.41–1.11]	0.61 ± 0.24 [0.29–1.19]	0.71 ± 0.20 [0.31–0.93]	1.15 ± 0.83 [0.21–6.21]	BDL	1.59 ± 3.02 [0–9.80]	0.40	*F*	2.24	0.24	2.16
										*P*-value	0.139	0.871	0.100
Na	0.44 ± 0.34 [0.13–1.10]	0.37 ± 0.24 [0.11–0.86]	0.71 ± 0.43 [0.09–1.36]	0.57 ± 0.26 [0.05–0.85]	0.72 ± 0.32 [0.38–1.20]	0.72 ± 0.54 [0.09–2.08]	0.84 ± 0.64 [0.09–1.53]	0.39 ± 0.21 [0.15–0.77]	0.13	*F*	3.43	2.22	1.20
										*P*-value	0.068	0.093	0.316
Mg	0.39 ± 0.29^a^ [0.09–0.91]	0.35 ± 0.22^a^ [0.06–0.66]	0.71 ± 0.26^bc^ [0.08–0.32]	0.45 ± 0.19^ab^ [0.03–0.68]	1.54 ± 0.51^d^ [1.13–2.36]	0.75 ± 0.28^bc^ [0.39–1.20]	1.62 ± 0.41^d^ [1.11–2.25]	0.98 ± 0.31^c^ [0.03–1.37]	0.10	*F*	35.20	37.20	5.60
										*P*-value	0.000*	0.000*	0.002*
P	0.80 ± 0.32 [0.20–1.31	0.57 ± 0.18 [0.20–1.31]	0.82 ± 0.34 [0.44–1.58]	0.48 ± 0.16 [0.30–0.81]	0.56 ± 0.14 [0.29–0.68]	0.48 ± 0.47 [0.14–1.65]	0.34 ± 0.28 [0.00–0.77]	0.49 ± 0.21 [0.24–0.86]	0.09	*F*	4.12	3.94	2.81
										*P*-value	0.046	0.012	0.045*
S	0.60 ± 0.26^bcd^ [0.14–0.98]	0.26 ± 0.07^a^ [0.19–0.36]	0.80 ± 0.38^d^ [0.41–1.60]	0.36 ± 0.10^ab^ [0.26–0.57]	0.73 ± 0.21^ cd^ [0.43–1.02]	0.48 ± 0.38^abc^ [0.22–1.44]	0.81 ± 0.21^d^ [0.53–1.12]	0.62 ± 0.43^bcd^ [0.09–1.63]	0.09	*F*	22.81	3.35	0.75
										*P*-value	0.000*	0.024*	0.524
Si	0.56 ± 0.29^bc^ 0.10–0.94]	0.49 ± 0.16^bc^ [0.29–0.76]	0.67 ± 0.45^c^ [0.04–1.45]	0.41 ± 0.09^bc^ [0.27–0.58]	0.38 ± 0.33^b^ [0.00–0.72]	0.57 ± 0.39^bc^ [0.07–1.55]	BDL	0.41 ± 0.31^bc^ [0.00–0.85]	0.09	*F*	1.15	5.80	5.29
										*P-*value	0.288	0.001*	0.002*
Fe	0.13 ± 0.24 [0.00–0.80]	0.02 ± 0.03 [0.0–0.08]	0.04 ± 0.03 [0.00–0.10]	0.04 ± 0.02 [0.0–0.08]	0.03 ± 0.01 [0.02–0.06]	0.23 ± 0.60 [0.0–1.93]	0.05 ± 0.02 [0.03–0.10]	0.11 ± 0.12 [0.00–0.44]	0.07	*F*	0.52	0.55	0.65
										*P*-value	0.475	0.647	0.236
K	0.07 ± 0.05^abcd^ [0.01–0.16]	0.04 ± 0.02^abc^ [0.00–0.06]	0.14 ± 0.21^d^ [0.00–0.51]	0.03 ± 0.02^ab^ [0.01–0.06]	0.03 ± 0.01^ab^ [0.00–0.04]	0.10 ± 0.10^d^ [0.00–0.32]	BDL	0.13 ± 0.13^ cd^ [0.01–0.31]	0.03	*F*	0.55	0.37	6.25
										*P*-value	0.461	0.773	0.001*
Zn	0.01 ± 0.02 [0.00–0.05]	BDL	0.08 ± 0.07 [0.00–0.16]	0.04 ± 0.04 [0.00–0.10]	BDL	BDL	0.04 ± 0.03 [0.00–0.09]	0.08 ± 0.08 [0.00–0.28]	0.02	*F*	n.a.	n.a.	n.a.
										*P*-value	n.a.	n.a.	n.a.
Mn	0.02 ± 0.06 [0.00–0.20]	BDL	0.01 ± 0.01 [0.00–0.02]	BDL	BDL	BDL	BDL	0.06 ± 0.12 [0.00–0.40]	0.02	*F*	n.a.	n.a.	n.a.
										*P*-value	n.a.	n.a.	n.a.
Cu	BDL	BDL	BDL	BDL	BDL	BDL	0.02 ± 0.04 [0.00–0.10]	0.08 ± 0.11 [0.00–0.30]	0.01	*F*	n.a	n.a	n.a
										*P*-value	n.a	n.a	n.a

Values are means, errors, and lower and upper quartiles (in brackets) for 10 eggshells of blackbird and 10 eggshells of song thrush (each for different egg).

Statistically significant main effects are shown in bold and marked with *(*P* < 0.05).

^a,b,c,d^No common superscript in a row indicates a statistical difference in elemental content in the shell layer.

*BDL* all samples below the detection limit.

*n.a.* not analyzed.

PCA was carried out to determine whether the differences observed between the eggshell layers of blackbird and song thrush were caused by the changes in elemental composition (Fig. 7C). This type of analysis allows visual interpretation of the relationships between variables (content of elements) and eggshell layers (Fig. 7C) and provides an overview of the impact of measured variables on the similarity or lack thereof between the analyzed samples. The results of the analysis showed that only Cr of blackbird and song thrush eggs was located closely on the PCA diagram, which means that this layer had a similar elemental composition. Moreover, Pa of blackbird eggshell had a similar elemental composition as Cr. The remaining eggshell layers differed in their compositions.

## Discussion

The most important part of this study is that we analyzed the microstructure of blackbird and song thrush eggshells and compared it with the data of other avian species. Our main focus was the elongated Mk, or the calcite cones surrounding them, forming a large space between white-side membrane fibers (smf) and Ma, which has not been investigated in most studies analyzing the morphology of avian eggshells. However, according to the SEM analysis of chicken eggshells in earlier studies^4,20^ and analyses of eggshells of other species whose eggs weigh greater than chicken eggs^21–24^, Mk are hidden within Ma without forming such structures or are markedly smaller. In contrast, the eggshell micrographs of zebra finch^22^ or American restart^25^, whose eggs weigh significantly lesser than blackbird and song thrush eggs, have shown similar structures^26^. Apart from these two recent studies on the eggshell morphology of small-sized eggs, most studies have only examined the shells of eggs with a high or very high weight, and the systematic impact^21–24^. Therefore, our study analyzed the microstructure of chicken and Chinese quail eggs (Fig. 3). The eggs of two species classified among Phasianidae, which are distinct from Turdidae, and Chinese quail have similar weight (5.6–7.1 g^27^) to blackbird and song thrush eggs (7.01 ± 0.17 and 6.06 ± 0.16, respectively^26^), while domestic chicken eggs are significantly heavier (about 50–60 g). This suggests that Mk of Chinese quail eggshell are more elongated and similar to those in blackbird and song thrush eggs, but not in the larger chicken eggs, although both species belong to the same family.

Another important observation is that the main structural units in the mineral layer of the eggshells of both analyzed species differ in shape compared to that in the eggshells of significantly larger eggs. Specifically, the entire units comprising mainly Cr and Pa are less prolonged and much wider than those in the eggshells of chicken^20^ or ratites^23^. We speculate that both elongated Mk, which form an “air duct” between smf and Ma, and the short and wide major eggshell units are evolutionary elements enabling gas exchange in eggs of lower weight. These structures limit water evaporation from the egg, both before and during incubation. Ar et al.^28^ observed in 29 species that lower egg weight correlated with lower gas conductance between the egg content and the environment. This finding is not related to the egg mass itself, but the different ratio of open pores found on the eggshell to its thickness. The vapor diffusion coefficient values determined for blackbird (1.95 mg/day^–1^/torr^–1^) and song thrush (1.83 mg/day^–1^/torr^–1^) eggs were similar to that calculated by Ar et al.^28^ for American robin (1.42 mg/day^–1^/torr^–1^), a closely related species that lays eggs of approximately 6.6 g. We also noted closed porosity on 3D micrographs (Fig. 5). Literature has no data regarding closed pores, i.e. pores whose channels run long the eggshell without any outlets. Although a discussion was not possible due to the lack of data, our observation suggests that further study is needed to confirm or exclude the presence of pores in the eggshell structure of birds other than blackbird and song thrush. However, the specificity of eggshell porosity is only one of the factors determining the dynamics of vapor diffusion through the eggshell. According to Booth and Rahn^37^, the intensity of this process also depends on the rate of eggshell thinning due to mineral loss during embryogenesis or pressure increase within the egg. Increased pressure results in increased rate of vapor diffusion during incubation. However, this phenomenon has been observed for domestic chicken and turkey, which lay relatively heavy eggs^29^. For sparrows, which lay eggs of very low weight, water diffusion from egg increases only till day 4 of incubation and then remains constant^30–33^. There is currently no information available to confirm whether this pattern of vapor diffusion from eggs is also valid for thrush. However, all avian embryos first deplete calcium from Mk, followed by other layers, when chorioallantois connects through the eggshell membrane with its outer surface^33^. Our observations show that the process is likely limited in the studied species due to the large calcium stocks in the elongated Mk and the natural space they form by separating the embryonic vessels from the Ma surface.

The European population studies have shown that blackbird is at least four times more numerous and has a greater reproductive success than song thrush whose number is declining^34^. The reason for this is unknown, but the present study indicates that it may be directly linked to the differences in eggshell morphology. The higher thickness of blackbird eggshell is likely due to the greater egg weight, but it is unclear why this results solely from the two thicker outermost layers. We may hypothesize that the thicker Cr and Pa in blackbird eggshell provide it with greater resistance to cracking under an external force, which may originate from a larger and harder egg of common cuckoo. Both blackbird and song thrush have adopted to protect their nests from brood parasitism during evolution. The larger blackbird can drive a common cuckoo away from the nest, whereas the smaller song thrush cannot. However, song thrush has developed a unique eggshell color and pigmentation pattern that allows it to easily detect and remove foreign eggs from nest^35^. Nonetheless, egg-laying by common cuckoo often leads to the cracking of one or more host eggs^36^. Therefore, the thicker Cr and Pa may be considered a protective factor and as contributing to the greater reproductive success of blackbird eggshell compared to song thrush. Protonmotoric pigment spots on the surface may have a similar function. Researchers have been investigating the function of eggshell pigment spots in recent years^37–39^. Apart from their protective role (camouflage) in the eggshells of birds building open nests, such as blackbird and song thrush, the “signaling function hypothesis” and “structural function hypothesis” suggest other functions for pigment spots. The first hypothesis refers to the quality of female and involvement of male in egg care^37^, while the second indicates that pigment spots intensify areas of reduced calcium saturation on eggshell, improving its durability^38,39^. Our results may contribute to expanding the knowledge about the structural function. We assume that the reproductive success of song thrush is lower, which may be partially because it invests a considerable amount of the blue eggshell pigment to protect its eggs from common cuckoo due to the reduced pigment spots on the egg surface. This allows song thrush to protect its eggs from being mimicked by brood parasites, but at the cost of their durability. On the other hand, increased distribution of pigment spots on the surface might be an evolutionary mechanism of blackbird to strengthen the eggshell. However, this should be confirmed by multigeneration studies on the evolution of the eggshell structure of both species and by simultaneous monitoring of their reproductive success.

## Conclusions and perspectives

Our study provides new information regarding avian eggshell structure, and it is the first to conduct an analysis of blackbird and song thrush eggshells. The findings presented here are of importance because it is difficult or even impossible to conduct studies on the eggs of numerous wild birds due to ethical reasons. At present, the size of blackbird and song thrush populations remains stable and therefore approval could be obtained for the collection of their eggs. Studies providing novel data on wild bird eggs may allow determining the variability of the discussed traits in the future meta-analyses for a greater number of birds classified as distinct species, families, and populations.

The present study analyzed several aspects related to the morphology, structure, chemical composition, and pigmentation of blackbird and song thrush eggshell. Two main observations were the differences in the morphology and pigmentation of the eggshells of these two species and the presence of highly elongated Mk in both. We established a hypothesis for each observation. According to the first hypothesis, the differences in pigment distribution and thickness of the outer eggshell layers in closely related bird species, inhabiting the same environment, might be an evolutionary adaptation to protect against brood parasitism. The second hypothesis is that the length of Mk is related to egg weight, and the lower the egg weight, the longer the Mk. This relationship is linked to the water diffusion coefficient of eggshell, and it has been shown that water diffusion prevents excessive water loss from low-weight eggs. Both hypotheses, but particularly the second one, should be confirmed by further research on eggshells of a large number of avian species characterized by highly diverse egg weight and breeding under different climatic conditions. Such research can be possible by making use of the museum collections of bird eggs. However, analysis of the structure, measurements of individual layers, and porosity of eggshell typically requires a small sample and observation of the cross-section surface. This may cause damages (fragmentation) in the eggshell, which is unacceptable in the case of museum collections. Therefore, further multidisciplinary research is recommended on the eggshells of wild birds, whose populations are stable enough to avoid any impact on their reproductive success. The results of our study utilizing modern measurement techniques may allow for repeated analysis and discussion of different researchers in the fields of biology, evolution, and ecology of birds.

## Materials and methods

### Ethics statement

All procedures related to the acquisition of blackbird and song thrush eggs from natural sites, as well as their temporary detention, transport, and dismantling for collecting study material, were approved by the Regional Director of Environmental Protection in Warsaw (PL) (12 April 2018, WPN-I.6401.102.2018.KZ.2).

All study procedures were performed in accordance with the guidelines of the Third Local Ethics Committee on Animal Experimentation in Warsaw (SGGW). As analyses were carried out on nonincubated eggs, our experiment did not require the direct consent of the National Ethical Commission at the Ministry of Science and Higher Education in Poland (Directive 2015/266/EC; Public Information Bulletin, 2017)^40^.

### Eggs and eggshell collection

A total of 10 eggs of blackbird (*T. merula*) and song thrush (*T. philomelos*) each were obtained. Each egg originated from a different female. The eggs were collected before the commencement of their incubation (female brooding)—about 1–3 days from laying. After collection eggs from nests and transport to the Institute of Animal Science laboratory (Warsaw, PL), which lasted for about 40 min, the eggs were placed in a cold store at 8 °C for 24 h. Then all eggs were broken, and the eggshells were washed in deionized water, dried at room temperature (about 23 °C) for another 24 h and analyzed successively. The specific procedures used for collecting eggs from nests, their transport, and storage were previously described in detail by Damaziak et al.^26^.

### SEM analysis

Eggs were cut in the middle portion of the long axis, and approximately 3 × 3 mm samples were taken toward the blunt end. The eggshell cuttings were installed on micro- to nanotables, in two planes (transversal to observe cross section of eggshell and with outer surface up to observe pigment spots), and were sputtered with gold (200 Å) using Cressington 108 auto/SE (Watford, UK). To observe smf from the sputtered eggshell samples, smf patches were partially separated, leaving some portion of the eggshell intact, enabling their installation for observation in transversal position. The patches were observed under a Phenom ProX scanning electron microscope (Phenom-World B.V., FEI Company, Eindhoven, the Netherlands), equipped with an electron beam, at an accelerating voltage of 5.0–15.0 kV, resolution of 14 nm, and a very wide electron-optic magnification (up to 150,000×). SEM micrographs were obtained for two samples from each of the 10 blackbird and song thrush eggs, and two samples from two eggs of Chinese quail (*Synoicus chinensis*) and domestic chicken (*Gallus gallus*) (commercial laying ISA Brown hens). The eggshell micrographs were used for comparative structural analysis of the avian species, whereas smf micrographs were used for the measurement of shell-side and white-side membrane fibers. In each analyzed sample (n = 10 for blackbird and n = 10 for song thrush), 10 fibers for each membrane were measured using FiberMetric Desktop SEM (Phenom-World B.V., FEI Company, Eindhoven, the Netherlands). Binarization of Smf micrographs (white side and shell side membrane)^41^ was performed using thresholding, where the Otsu method^42^ was used to determine the threshold value. Pixels lighter than the threshold value were assigned a value of zero (fiber) and darker pixels were assigned a value of 1 (background). The computer image analysis software MultiScanBase v. 18.03 was used.

The actual pigment color of the eggshells was visualized using VHS Digital Microscope (Keyence, Belgium) to observe irregular surfaces and three-dimensional (3D) objects. Photographs were taken at a magnification of 20 ×.

### X-ray computed microtomography

For scanning, eggshell cuttings (5 × 5 mm) were collected from the egg equator line. A total of 16 samples were prepared for the analysis (8 for song thrush and 8 for blackbird; 2 from 4 eggs of each species). The samples were placed on a round plate. The source and detector were fixed, while the sample was rotated during measurement. All scans were performed using a SkyScan1172 desktop microCT scanner (Bruker-microCT, Kontich, Belgium), applying a 180° scan rotation with 0.4° rotation step and × 4 frame averaging (except where stated otherwise). The source–object–camera distance was adjusted to obtain images with a pixel size of 5 µm. Radiation of 40 kV and 193 µA without filter was applied. To record cone transmission of X-ray beam, a CCD camera with a resolution of 11 MP was used. The duration of one scan was 39 min.

Flat images of cross sections of each sample were obtained following tomographic reconstruction by NRecon 1.6.9.8 software (Bruker-microCT, Kontich, Belgium). A 3D grayscale datastack was created, which was typically digitized to 137 slices. Gaussian smoothing and ring artifact reduction correction were applied.

For image analysis, the datastack margins were cut to a rectangle measuring 20 × 10 pixels (1 pixel = 5 µm) using CTAn 1.9.3.3 software (Bruker-microCT, Kontich, Belgium) (Fig. S1). The series of two-dimensional (2D) slices were reconstructed into a 3D image. Then, 3D objects with a VOI of 3.42 × 10^–3^ voxels were measured. The following 3D parameters were calculated (Skyscan 2008)^43^: (i) object volume (total volume of binarized objects within the VOI), (ii) percent object volume (the volume of all solid objects within the VOI ratio of pore surface area to object volume), (iii) object surface/volume ratio (the surface area of all eggshell structures divided by the total volume of the eggshell within the analyzed volume; the ratio of solid surface to volume measured in 3D within the VOI, which is a useful parameter for characterizing the thickness and complexity of structures), (iv) object surface (the surface area of all solid objects within the VOI), (v) object surface density (the ratio of surface area to total volume measured as described for 3D, within the VOI), vi) Euler number (complex 3D structure connection indicator, and a global parameter of topology; it denotes the number of connections in a structure that can be severed before the structure falls into two separate pieces; high Eu values indicate poorly connected structures), and (vii) fractal dimension (an indicator of surface complexity of an object, which quantifies how that object’s surface fills space). CTvol software was used to render the reconstructed cross sections by volume in order to achieve a realistic visualization of the reconstructed eggshell microstructure.

### Morphometric water vapor conductance (G_H2O_)

Morphometric water vapor conductance (G_H2O_) was calculated in the eggshells of blackbird and song thrush using the formula of Tanaka et al.^44^: G_H2O_ = 2.1 Ap/Ls.

In 664 photos of the 2D eggshell microstructure obtained using microtomograph, CTAn software marked a section with a surface area of 20 × 10 pixels (1 pixel = 5 µm). Then, the number of pores (n), average object area-equivalent circle diameter (mm), and mean individual pore area (mm^2^) were calculated.

The eggshell surface area A_s_ (cm^2^) was calculated using the formula of Paganelli et al.^45^: A_s_ = 4.835·M^0.666^, where M denotes egg weight. Egg weight measured by Damaziak et al.^26^ was used for the calculations.

Total pore number (N) in the eggshell was determined by multiplying the number of pores (n) by eggshell surface area (A_s_) and 2D image surface area.

Eggshell porosity (A_p_/L_s_ (mm)) was calculated by dividing the total egg pore surface area (A_p_ (mm^2^)) by pore length (L_s_ (mm)), where total pore area (A_p_ (mm^2^)) was determined by multiplying total pore number (N) by mean individual pore area (A (mm^2^)). Based on the 2D microstructure photographs, shell thickness (L_s_ (µm)) was determined.

### Mineral composition of eggshell

Mineral composition was determined by scanning electron microscopy–energy-dispersive X-ray spectroscopy (SEM–EDS) using an energy dispersion spectrometer (SDD type) integrated with a scanning electron microscope (Phenom-World B.V., FEI Company, Eindhoven, the Netherlands). For each examined eggshell sample (2 samples × 10 eggshells × 2 species), the analysis was carried out in two randomly selected points on each of the four layers (Cr, Pa, Ma, Mk) as presented in Fig. 7.

### Statistical analysis

Statistical analysis was performed using Statistica 13 PL software. The variance of the data are expressed as mean values ± standard deviations. To establish the differences between the samples analyzed, a one-factor analysis of variance (ANOVA) was performed for a significance level α < 0.05 using Duncan’s Multiple Range test. In addition, principal component analysis (PCA) was performed.

## Supplementary Information

Supplementary Figure S1.

## Data Availability

The datasets analyzed in the current study are available from the corresponding author on reasonable request.
