# Supplementary material for: Analysis of ultrastructure and microstructure of blackbird (Turdus merula) and song thrush (Turdus philomelos) eggshell by scanning electron microscopy and X-ray computed microtomography
Source: Sci Rep. 2022 Jul 13;12:11857. doi: 10.1038/s41598-022-16033-5 (PMC9279409; doi:10.1038/s41598-022-16033-5)

**Ultrastructure and microstructure of blackbird (*Turdus merula*) and song thrush (*Turdus philomelos*) eggshell observed with scanning microscope and using X-ray computed microtomography**

**Krzysztof Damaziak & Agata Marzec**

**Supplementary Figure S1. ROI (10 × 20 pixels) view used for blackbird (*Turdus merula*) and song thrush (*Turdus philomelos*) eggshell microstructure D2 measurements.**


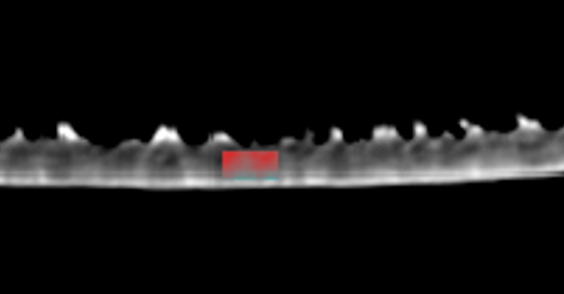


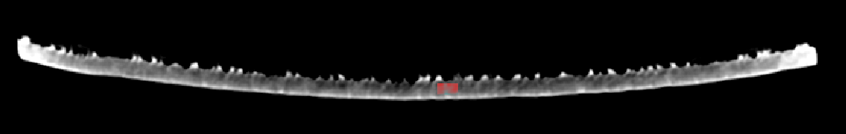

Supplement: Supplementary file 1 — Supplementary Figure S1. [file 41598_2022_16033_MOESM1_ESM.docx]
